# Identification of Chemical Profiles and Biological Properties of *Rhizophora racemosa* G. Mey. Extracts Obtained by Different Methods and Solvents

**DOI:** 10.3390/antiox9060533

**Published:** 2020-06-18

**Authors:** Annalisa Chiavaroli, Koaudio Ibrahime Sinan, Gokhan Zengin, Mohamad Fawzi Mahomoodally, Nabeelah Bibi Sadeer, Ouattara Katinan Etienne, Zoltán Cziáky, József Jekő, Jasmina Glamočlija, Marina Soković, Lucia Recinella, Luigi Brunetti, Sheila Leone, Hassan H. Abdallah, Paola Angelini, Giancarlo Angeles Flores, Roberto Venanzoni, Luigi Menghini, Giustino Orlando, Claudio Ferrante

**Affiliations:** 1 Department of Pharmacy, University “G. d’Annunzio” of Chieti-Pescara, 66100 Chieti, Italy; annalisa.chiavaroli@unich.it (A.C.); lucia.recinella@unich.it (L.R.); luigi.brunetti@unich.it (L.B.); sheila.leone@unich.it (S.L.); luigi.menghini@unich.it (L.M.); claudio.ferrante@unich.it (C.F.); 2 Department of Biology, Science Faculty, Selcuk Universtiy, Campus Konya, 42130 Konya, Turkey; sinankouadio@gmail.com; 3 Institute of Research and Development, Duy Tan University, Da Nang 550000, Vietnam; mohamadfawzimahomoodally@duytan.edu.vn or; 4 Department of Health Sciences, Faculty of Science, University of Mauritius, Réduit 230, Mauritius; nabeelah.sadeer1@umail.uom.ac.mu; 5 Laboratoire de Botanique, UFR Biosciences, Université Félix Houphouët-Boigny, 01 Abidjan, Ivory Coast; katinan.etienne@gmail.com; 6 Agricultural and Molecular Research and Service Institute, University of Nyíregyháza, 4400 Nyíregyháza, Hungary; cziaky.zoltan@nye.hu (Z.C.); jjozsi@gmail.com (J.J.); 7 Department of Plant Physiology, Institute for Biological Research “Siniša Stanković”, University of Belgrade, 11000 Belgrade, Serbia; jasna@ibiss.bg.ac.rs (J.G.) marina.sokovic@mpn.gov.rs (M.S.); 8 Chemistry Department, College of Education, Salahaddin University-Erbil, Erbil 44001, Iraq; hassan.abdullah@su.edu.krd; 9 School of Pharmaceutical Sciences, Universiti Sains Malaysia, USM, Penang 11800, Malaysia; 10 Department of Chemistry, Biology and Biotechnology, University of Perugia, 06100 Perugia, Italy; giancarlo.angelesflore@studenti.unipg.it (G.A.F.); roberto.venanzoni@unipg.it (R.V.)

**Keywords:** mangrove, phytochemical, homogenizer-assisted extraction, phytoanti-oxidants, enzyme inhibition, anti-microbial activity, in silico studies

## Abstract

Mangrove forests exemplify a multifaceted ecosystem since they do not only play a crucial ecological role but also possess medicinal properties. Methanolic, ethyl acetate and aqueous leaf and bark extracts were prepared using homogenizer-assisted extraction (HAE), infusion and maceration (with and without stirring). The different extracts were screened for phytochemical profiling and antioxidant capacities in terms of radical scavenging (DPPH, ABTS), reducing potential (CUPRAC, FRAP), total antioxidant capacity and chelating power. Additionally, *R. racemosa* was evaluated for its anti-diabetic (α-amylase, α-glucosidase), anti-tyrosinase and anti-cholinesterase (AChE, BChE) activities. Additionally, antimycotic and antibacterial effects were investigated against *Eescherichia coli, Pseudomonas aeruginosa, Salmonella typhimurium, Listeria monocytogenes, Enterobacter cloacae, Bacillus cereus, Micrococcus luteus, Staphylococcus aureus, Aspergillus fumigatus, Aspergillus niger, Trichoderma viride, Penicillium funiculosum, Penicillium ochrochloron* and *Penicillium verrucosum*. Finally, based on phytochemical fingerprint, in silico studies, including bioinformatics, network pharmacology and docking approaches were conducted to predict the putative targets, namely tyrosinase, lanosterol-14-α-demethylase and *E. coli* DNA gyrase, underlying the observed bio-pharmacological and microbiological effects. The methanolic leave and bark extracts (prepared by both HAE and maceration) abounded with phenolics, flavonoids, phenolic acids and flavonols. Results displayed that both methanolic leaf and bark extracts (prepared by HAE) exhibited the highest radical scavenging, reducing potential and total antioxidant capacity. Furthermore, our findings showed that the highest enzymatic inhibitory activity recorded was with the tyrosinase enzyme. In this context, bioinformatics analysis predicted putative interactions between tyrosinase and multiple secondary metabolites including apigenin, luteolin, vitexin, isovitexin, procyanidin B, quercetin and methoxy-trihydroxyflavone. The same compounds were also docked against lanosterol-14α-demethylase and *E. Coli* DNA gyrase, yielding affinities in the submicromolar–micromolar range that further support the observed anti-microbial effects exerted by the extracts. In conclusion, extracts of *R. racemosa* may be considered as novel sources of phytoanti-oxidants and enzyme inhibitors that can be exploited as future first-line pharmacophores.

## 1. Introduction

Mangrove forests are the most productive and richest ecosystems on the planet. They provide valuable ecological benefits to the coastal lines of tropical and sub-tropical countries by acting as a protective barrier against waves, purify the nearby marine environment and provide habitats for countless reptiles, birds and fish [1]. Besides their perennial ecological roles, mangroves hold another important facet, which we sometimes overlook—the therapeutic value possessed by the plants. The intuitive nature of humans believed that the different parts of mangroves can cure a variety of diseases, namely rheumatism, diabetes, snake bites, asthma, skin diseases, throat pains, diarrhea, fever, intestinal worms, among others [2,3]. However, most of these traditional beliefs have not been verified yet, which subsequently triggers a dire need for a thorough investigation. Indeed, as supported by one of our recent comprehensive review on mangroves, it is mentioned that although a total of 84 mangrove species are recorded to-date, only 27 of them have been validated for their pharmaceutical effects [4]. 

This considerable meagerness of studies on the pharmacological aspects of mangroves has not changed during the past decades and it is evident by a lack of published work regarding this matter. Therefore, to fill this niche, we focus our work on an untapped mangrove species named *Rhizophora racemosa* G. Mey. (Family: Rhizophoraceae) by evaluating its pharmacological properties and identifying phytochemicals present. Morphologically, *R. racemosa* is a tree reaching a height of up to 30 m developing stilt roots and elliptical leaves. This species has the potential to bloom 128 flowers on one axillary branch. Sepals of flowers are 8–10 mm long [5]. The Nigerian people traditionally used the leaves of *R. racemosa* to treat toothache and dysmenorrhea. A study was conducted to determine the lethal dose (LD50) of the methanolic leaf extract. Results showed that the LD50 of the extract was 1583.33 mg/kg which is considered safe for consumption [6]. In Benin, the roots of the mangrove plant, locally called Wéto, is used to manage malaria [7]. 

In a revision of phytochemical knowledge of the genus [8], it is reported that the species *R. racemosa* from Australia contains primary and secondary metabolites, such as sugars and polysaccharides, aminoacids as well as polyphenols, triterpenes and tannins. The phytochemical profile of water extract from the bark of *R. racemosa* includes saponins and terpenes and possesses anti-microbial activity against the phytopathogen fungus *Lasiodiplodia theobromae* [9]. The presence of multiple classes of secondary metabolites was qualitatively confirmed [10].

Out of the seven accepted members of the *Rhizophora* genus [11], *R. racemosa* is considered as underexplored since as far as our literature could establish, we noticed a dearth of information with no attempt in evaluating the antioxidant properties and enzymatic inhibitory effects of that particular mangrove species. Thus, our present study aims at shedding more light on this poorly understood plant. Our work is as follows: (1) characterize the phytochemicals present in leaves and barks using in vitro standard assays, (2) assess antioxidant properties in terms of radical scavenging, reducing potential, total antioxidant capacity and metal chelating, (3) evaluate the enzymatic inhibitory effects related to chronic diseases namely diabetes mellitus type II, neurodegenerative complications and skin disorders and (4) make a comparison between different extraction methods (infusion, homogenizer assisted extraction, maceration (with and without stirring)) used to prepare the samples. Additionally, antimycotic and antibacterial effects were investigated against *Escherichia coli, Pseudomonas aeruginosa, Salmonella typhimurium, Listeria monocytogenes, Enterobacter cloacae, Bacillus cereus, Micrococcus luteus, Staphylococcus aureus, Aspergillus fumigatus, Aspergillus niger, Trichoderma viride, Penicillium funiculosum, Penicillium ochrochloron* and *Penicillium verrucosum*. Finally, based on phytochemical fingerprint, in silico studies, including bioinformatics, network pharmacology and docking approaches were conducted to predict the putative targets underlying the observed bio-pharmacological and microbiological effects.

## 2. Materials and Methods 

### 2.1. Plant Material and Preparation of Extracts

*R. racemosa* was collected in Mafiblé village, municipality of Port-Bouët, city of Abidjan of Côte d’Ivoire, in the year 2019 and it was authenticated by the botanist Ouattara Katinan Etienne (Université Félix Houphouet Boigny, Abidjan, Côte d’Ivoire). The plant materials were dried in the shade in an air-ventilated environment (about 10 days). The leaves and stem barks were carefully separated. These plant materials were powdered by using a laboratory mill. The powdered plant materials were stored in the dark at 20 °C. 

**Figure 1:**
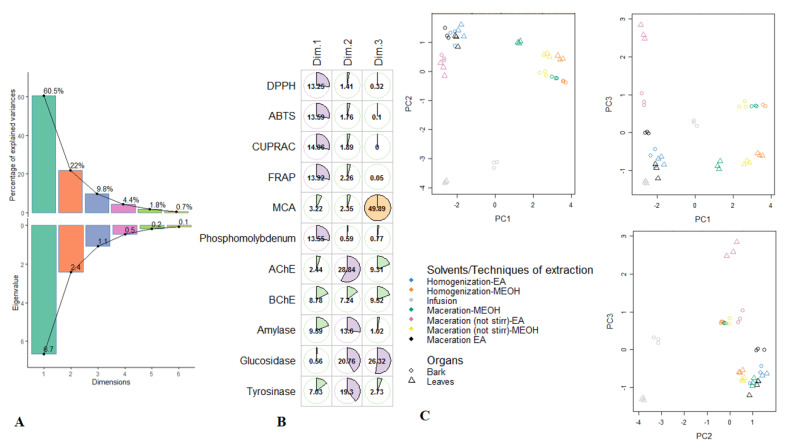
Principal component analysis on *R. racemose* biological activities. (**A**). Percentage of variability and eigenvalue explained by each principal component. (**B**). Relation between the biological activities and the first three principal components. (**C**). Samples plots.

We used homogenizer-assist extraction (HAE), maceration (with stirred and without stirred) and infusion techniques in the present study. Ethyl acetate and methanol were used for HAE and maceration techniques. Water was used in the infusions. The flowchart is given in Figure 1. Obtained extracts were filtered and then evaporated by using a rotary evaporator. Infusions were lyophilized and all dried extracts were stored at 4 °C until analysis. 

### 2.2. Profile of Bioactive Compounds

The total phenolic, phenolic acid, flavanol and flavonoid contents of the extracts were measured and detailed methods were described in our previous paper [12,13]. Standards, namely gallic acid (GAE) for phenolics, caffeic acid (CE) for phenolic acid, catechin (CAE) for flavanol and rutin (RE) for flavonoids, were used to explain the results. 

Gradient reversed-phase ultra high performance liquid chromatography (UHPLC) separations with electrospray MS/MS detection (both positive and negative ion modes) were used for the structural characterization of the compounds presenting in different extracts. The UHPLC system consisted of the Dionex Ultimate (Thermo Scientific, Waltham, MA, USA) 3000RS UHPLC instrument coupled to a Thermo Q Exactive Orbitrap mass spectrometer (Thermo Scientific, Waltham, MA, USA). Chromatographic separation was achieved on a reversed-phase column Thermo Accucore (Thermo Scientific, Waltham, MA, USA) C18 (100 mm × 2.1 mm i. d., 2.6 μm) [14]. All analytical details were given in Supplementary Material.

### 2.3. Determination of Antioxidant and Enzyme Inhibitory Effects

To detect antioxidant properties, we used several chemical assays including different mechanisms namely, radical scavenging ((2,2′-azino-bis (3-ethylbenzothiazoline-6-sulphonic acid) (ABTS), and 2,2-diphenyl-1-picrylhydrazyl (DPPH)), reducing power (ferric reducing power (FRAP, transformation from Fe^3+^ to Fe^2+^ by anti-oxidants), cupric reducing power (CUPRAC, transformation from Cu^2+^ to Cu^+^ by anti-oxidants)), phosphomolybdenum and metal chelating. Trolox (TE) and ethylenediaminetetraacetic acid (EDTA) were used as standard antioxidant compounds. Obtained results were expressed as equivalents of the standard compounds [15]. All experimental details were given in Supplemental Materials.

To detect inhibitory effects on enzymes, we used colorimetric enzyme inhibition assays and these assays included tyrosinase, α-glucosidase, α-amylase, and cholinesterases (acetylcholinesterase (AChE), butyrylcholinesterase (BChE)). Some standard inhibitors (galantamine for cholinesterases; kojic acid for tyrosinase and acarbose for α-glucosidase, α-amylase) were used as positive controls. The results were expressed as equivalents of the standard compounds. All experimental details were given in Supplemental Materials.

### 2.4. Antibacterial and Anti-fungal Activities

The Microdilution method was used to evaluate antibacterial and anti-fungal properties of the extracts following methods described in our earlier study [16]. *Staphylococcus aureus* (ATCC (American Type Culture Collection, Manassas, VA, USA) 6538), *Listeria monocytogenes* (NCTC 7973), and *Bacillus cereus* (clinical isolate) were used as Gram-positive bacteria. *Salmonella typhimurium* (ATCC 13311), *Pseudomonas aeruginosa* (ATCC 27853), *Enterobacter cloacae* (human isolate), and *Escherichia coli* (ATCC 35210) were used as Gram-negative bacteria.

Fungi, namely, Aspergillus fumigatus (human isolate), Aspergillus ochraceus (ATCC 12066), Aspergillus niger (ATCC 6275), Aspergillus versicolor (ATCC 11730), Trichoderma viride (IAM 5061), Penicillium funiculosum (ATCC 36839), Penicillium ochrochloron (ATCC 9112) and Penicillium verrucosum var. cyclopium (food isolate), were used to investigate the anti-fungal properties of the extracts.

Anti-microbial results were evaluated by minimum inhibitory (MIC) and minimum bactericidal/fungicidal (MBC/MFC) concentrations. Ampicillin and Streptomycin were used as standards for antibacterial activity. Bifonazole and ketoconazole were used as positive controls for anti-fungal evaluation.

### 2.5. Bioinformatics and Docking Studies

Chemical structures were prepared and converted into canonical. The simplified molecular-input line-entry system (SMILES) using ChemSketch software (Advanced Chemistry Development, Inc., (ACD/Labs), Toronto, ON, Canada). The SMILES were then processed by the SwissTargetPrediction (http://www.swisstargetprediction.ch/) platform, for predicting putative protein targets. The names of the identified targets were normalized according to the UniProt database (https://www.uniprot.org/). Cytoscape software (3.7.2 version; National Institute of General Medical Sciences (NIGMS), Bethesda, MD, USA) was used to create a Venn diagram of identified phytochemicals in the tested extracts and a components-targets illustration network.

Regarding the docking analysis, the routine steps for docking calculations involve the preparation of the inhibitors and the protein. The crystal structures of the proteins were downloaded from Protein Data Bank (PDB). The PDB codes were: 5M8P; 6RKS; 4LXJ. To prepare the protein for docking calculations, all water molecules and co-crystallized compounds were removed. This step was followed by adding polar hydrogen atoms and neutralized using the Autodock4 program (Molinspiration Database). The starting structures of secondary metabolites were optimized to their ground state structures using the AM1 semiempirical method and the 3D structures were saved in mol2 format. The protein was immersed in a 3D grid box with 60 × 60 × 60 dimensions with 0.375 Å distance between points. A Lamarckian genetic algorithm was used to calculate the docking free energy of 250 confirmations for each inhibitor. The docking results were clustered and organized according to the docking free energy. The binding site was localized, and the non-bonding interactions were elucidated using Discovery Studio 5.0 visualizer.

### 2.6. Statistical Analysis

Results were done as a mean ± standard deviation (SD). One-way analysis of the variance was used to evaluate significant differences among samples (*p* < 0.05, Turkey’s post hoc test) for each assay done. Principal component analysis was performed to visualize the (dis)similarity between the samples and k-medoids cluster analysis was used for the classification. The performance of k-medoids clustering was assessed by estimating the silhouette coefficient (*S_i_*). A value of *S_i_* close to 1 indicates a good clustered. 

The dataset was submitted to Supervised PLS-DA (Sparse Partial Least Squares) in an attempt to discriminate *R. racemosa* organs. Model goodness was recorded by estimating area under curve (AUC) value. Variable importance in projection was achieved to determine the influence of each biological activity in organ separation. Biological activities with variable importance of projection (VIP score) > 1.1 were considered to have the highest discrimination potential (VIP score > 1). R v 3.6.1 statistical software was used for the analysis (R Core Team, Vienna, Austria). 

## 3. Results 

### 3.1. Profiling of Bioactive Compounds

Phenolic compounds are crucial for the physiology of both plants and human beings since they provide a protective role in the human body by acting as anti-oxidants against oxidative stress [17]. Furthermore, literature previously reported important biological activities, namely anti-cancer, anti-inflammatory, anti-microbial, anti-diabetic and neuroprotective [18,19,20]. Therefore, the quantitative estimation of phytochemicals of different extracts of the leaves and barks of *R. racemosa* was undertaken. The results are summarized in Table 1. Three different extraction techniques, namely infusion, maceration and homogenizer-assisted extraction (HAE) were employed to extract potential bioactive compounds. Among the aforementioned techniques, HAE is the most recommended one since it consumes a low amount of solvent and time and is environment friendly [21]. Indeed, our results revealed that the methanolic leaf extracts prepared by HAE yielded higher phenolic, phenolic acids and flavonol contents (217.21 mg GAE/g, 58.50 mg CAE/g and 62.43 mg CE/g, respectively). Likewise, the same technique extracted the highest quantity of phenolic and flavonol contents (210.00 mg GAE/g and 66.17 mg CE/g, respectively) from the methanolic bark extract. In terms of flavonoid extraction, infusion was the best technique yielding the highest amount (15.01 mg RE/g) from methanolic bark while maceration method extracted the highest amount from methanolic leaf extract (38.45 mg RE/g). Irrespective of the extraction methods used, leaf possessed the highest amount of phenolic and flavonoid in contrast to bark. The bark yielded the highest amount of phenolic acid and flavonol compared to leaf (Table 1).

The identification of compounds was based on their retention time, the accurate mass and the registered mass spectra fragmentation patterns. The UHPLC method used was very versatile and allows for the separation of components in the medium to low polarity range, however, it had limited resolving power for polar compounds (e.g., sugars, short-chain amino acids and organic acids), which generally elute in the void volume. A detailed list for chemical profiles is given in Supplemental Materials. 

Phenolic acids, flavonoids, di-, tri- tetra- and pentahydroxyflavone and flavanone derivatives, procyanidins and catechins were mostly identified in the extracts. Finally, 38–60 compounds were characterized in the extracts by comparing our LC-MS/MS data with data from the literature and with our previous results. We could identify a wide range of compounds e.g., lower molecular weight polar component (hydroxybenzaldehyde, molecular weight (MW): 122) or a catechin trimer (MW: 866) or flavon and flavanone derivatives (MW: 4–600). The most 56/60 compounds were detected, as expected, in methanol extracts, while the least compound was detected in ethyl acetate extracts. The leaves and bark were found to have a similar qualitative composition independently from extraction methods.

Many quinic acid derivatives were observed in the extracts. Components with the retention time 10.24, and 14.88 were assigned as chlorogenic acid and chlorogenic acid isomer according to their [M+H]^+^ ions at *m/z* 355.1029 and they showed characteristic product ions. Chlorogenic acid was identified by comparison with an authentic standard. These compounds were detectable in both polarities. *4*-Coumaric acid was identified in the extracts in negative ion mode (18.54 min, *m/z* 163.03952), by comparisons with a reference standard (Table 2).

Flavanol monomers catechin/epicatechin (retention time 14.00/17.61 min) were identified by molecular ion [M−H]^−^ *m/z* 289.07121 and their fragment ions. Catechin dimer, procyanidin B, was detected at 15.69 min by molecular ion [M−H]^−^ *m/z* 577.1346 and catechin trimer, procyanidin C, was detected at 17.33 min by molecular ion [M−H]^−^ *m/z* 865.19799.

Most of the flavonoids were detected as glycoconjugates, such as hexose, pentose and dihexose. Characteristic mass losses of *C*- and *O*-glycosides were observed in their MS/MS spectra. 

Compounds with the retention time 19.30, 21.70, 22.71 and 25.44 min (C_24_H_20_O_9_), displaying the same MS fragmentation, were identified as cinchonain I isomers. Compounds with retention time 16.08, 17.04, 17.91 and 18.09 min (C_39_H_32_O_15_) shared the same MS fragmentation pattern, correspond to cinchonain II type compounds. We could detect cinchonain type compounds in all extracts by their deprotonated molecular ions, but we confirmed the presence of compounds and listed in the Tables if we could record at least five characteristic fragments.

### 3.2. Antioxidant Activities

The methanolic, ethyl acetate and aqueous extracts of the two plant parts (leaves and barks) were subjected to six different antioxidant assays. We screened our different extracts using multiple assays in terms of their radical scavenging ability (DPPH and ABTS), reducing potential (CUPRAC and FRAP), total antioxidant capacity and metal chelating power. The collected data are summarized in Table 3.

It is important to highlight that the methanolic leaf and bark extracts prepared from the extraction technique, HAE, revealed the highest antioxidant properties with DPPH, ABTS, CUPRAC, FRAP and phosphomolybdenum assays. On the other hand, ethyl acetate leaves and bark extracts prepared from maceration (without stir) possessed the most potent chelating power. For instance, with the DPPH assay which involved the reduction of a ferric-tripyridyl triazine complex to its colored ferrous form in the presence of anti-oxidants, the methanolic leaf and bark extracts showed highest DPPH radical scavenging ability (525.84 and 512.37 mg TE/g, respectively). A similar trend was observed with ABTS assay classifying both the methanolic leaf and bark extracts as the most potent ABTS radical scavengers (600.84 and 581.39 mg TE/g, respectively). 

Based on the results displayed in Table 3, it can be noticed that methanolic leaf and bark extracts (both prepared by HAE) exhibited relatively the same total antioxidant capacity (4.82 and 4.74 mmol/TE, respectively) followed by methanolic leaf and bark (prepared by maceration) (4.29 and 4.51 mmol/TE, respectively). Remarkable reducing potential was demonstrated by methanolic bark (HAE) (CUPRAC: 1129.33 and FRAP: 633.53 mg TE/g) followed by methanolic leaf(HAE) (CUPRAC: 1047.10 and FRAP: 544.76 mg TE/g). However, lower activities were reported with the ethyl acetate leaf and bark extracts prepared by maceration (without stirring). 

Chelation of transition metals is also considered as an evaluation of the antioxidant ability of a compound. Several lines of evidence reported that transition metals namely Fe^2+^ or Cu^2+^ are active redox metals and through Haber Weiss and Fenton reactions lead to the formation of ROS [22]. Evaluating the different extracts of *R. racemosa* showed us that the ethyl acetate leaves, and bark extracts prepared by maceration (without stirring) possessed the highest chelating power (29.39 and 24.93 mg EDTAE/g, respectively).

### 3.3. Enzymatic Inhibitory Effects

The results of enzyme inhibitory effects are given in Table 4. 

Herein, our findings demonstrated that the leaves extracts prepared from HAE (ethyl acetate and methanolic), maceration (ethyl acetate and methanolic) and maceration (with and without stirring) exhibited relatively the same AChE activities with galantamine equivalent ranging from 8.35 to 8.78 mg/g. Likewise, the same trend was observed with bark extract resulting in galantamine equivalent ranging from 8.04 to 8.76 mg/g. However, extracts (both leaf and bark) prepared from infusion projected the least AChE inhibitory effect (Table 4). In terms of BChE inhibition, a different trend was observed whereby several inactivities were even reported with a few extracts irrespective of the extraction technique used. The ethyl acetate leaf extract (maceration without stirring) exhibited the highest BChE activity (3.50 mg GALAE/g) while aqueous leaf extract displayed the lowest activity (0.91 mg GALAE/g). On the other hand, ethyl acetate bark extract (maceration with stirring) showed the highest BChE inhibitory effect (3.03 mg GALAE/g). 

Blemished skin, acne scars, age spots, melasma and post-inflammatory hyperpigmentation do not only occur during puberty but equally affect adults. These skin problems are caused by an increase in melanin production and the enzyme responsible is tyrosinase. Therefore, searching for tyrosinase inhibitors are highly recommended to fight against these skin disorders [23]. Herein, we screened our extracts for tyrosinase inhibitory effects. Results showed that methanolic leaf and bark both prepared by maceration displayed the highest tyrosinase inhibition (146.76 and 154.33 mg KAE/g, respectively). While the lowest activity was recorded with bark infusion (25.64 mg KAE/g), no activity was noted with leaf infusion (Table 4). 

Furthermore, the extracts of *R. racemosa* was screened for α-amylase and α-glucosidase inhibitory activities since diabetes mellitus type II (DMII) is a growing pandemic and people suffering from DMII has an increased risk in developing chronic macrovascular (cardiovascular disease) and microvascular (chronic kidney disease) complications [24]. Herein, the methanolic leaf and bark extracts (both prepared by HAE) exhibited the highest α-amylase inhibitory activity (1.24 and 1.27 mmol ACAE/g, respectively). However, a different trend was observed with α-glucosidase. For instance, both methanolic leaf extracts prepared by HAE and maceration significantly depressed α-glucosidase activity (21.17 mmol ACAE/g, respectively). However, several extracts especially bark extracts were reported inactivity against α-glucosidase. 

### 3.4. Exploratory Analysis, Classification and Discriminant Analysis

After the initial screening of antioxidant properties and enzymatic inhibitory effects in the different *R. racemosa* extracts, multivariate statistics were employed on the dataset to group and then discriminate samples. Firstly the exploratory analysis i.e., PCA was computed and results were reported in Figure 1. The initial 11 biological parameters had generated three principal components, representing 92.3% of the total variance; in consideration of the eigenvalues which were larger than 1 (Figure 1A). The PC1 was much positively described with FRAP, CUPRAC, DPPH, ABTS and PPBD and its total variance was 60.5% (Figure 1B). This reflects strong properties in the anti-oxidants of samples being the right side of PC1, including Mac-MeOH, HAE-MeOH and Mac-(no stirring)-MeOH extracts of both organs. PC2 accounted for 22% of the total variance, exhibited significant positive loadings of AChE, glucosidase and tyrosinase. This indicates that PC2 separated, on its positive side, the samples showing excellent inhibition against AChE, glucosidase and tyrosinase of those with lower inhibition activity on its negative side. Thus, ethyl acetate and methanolic extracts of leaves and stem bark obtained with maceration, homogenization and maceration (no stirring) exhibited better inhibition against AChE and tyrosinase. However, concerning glucosidase, better inhibition was achieved by the samples of both organs being of the left side of PC2 i.e., the ethyl acetate extracts obtained with maceration, homogenization and maceration (no stirring). The third component PC3, which summarized 9.8% of the data variance and discriminated samples according to their ability to chelate ferrous ion. Indeed the methanol extracts of leaves and stem bark derived from maceration (no stirring) showed a better ferrous ion chelating activity. Scores plots of the first three PC were depicted in Figure 1C; some groups can be visually distinguished. In particular, the methanol extracts of bark obtained with maceration (no stirring), maceration and homogenization were closed together. It was the same with the methanol extracts of leaves derived from homogenization and maceration (no stirring). Additionally, ethyl acetate extracts of both organs obtained using homogenization and maceration seemed to constitute the same group. 

**Figure 2:**
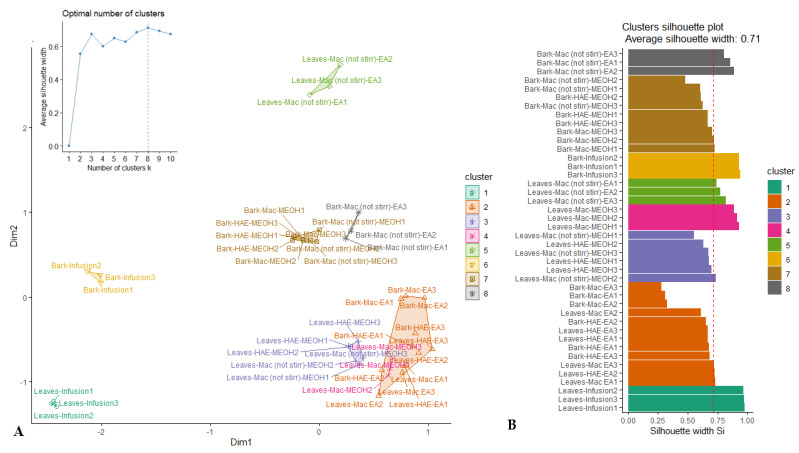
Cluster plot (**A**) and Silhouette plot (**B**) of K-medoids analysis.

PCA allowed for the highlighting of a similarity in biological activities among several samples of *R. racemosa.* However, some samples could not be assigned to a certain well-defined group. For this purpose, the k-medoids clustering statistic was employed under the result of PCA for classification of samples into a small number of homogeneous clusters. From the cluster map reported in Figure 2A, it can be seen that the samples were partitioned in eight clusters. Indeed, besides the three clusters emphasized through PCA, five other clusters were identified. The quality of the clustering was assessed using the average silhouette approach. As observed (Figure 2B), the clustering was excellent with average silhouette width of 0.71 which had revealed close to 1. 

Unsupervised multivariate analysis helped to spotlight a great variation among samples, which were consolidated into eight distinct clusters. However, an in-depth analysis provided a view of the aggregation of some samples according to the organs and/or the solvents/techniques of extraction. The extracts of the two organs had partnered in a distinct cluster, except their extracts obtained with HAE-EA and Mac-EA. In addition, while in the bark, Mac-MeOH, HAE-MeOH and Mac-(no stirring)-MeOH extracts were clustered together, in leaves, Mac-MeOH extracts differed from those of HAE-MeOH and Mac-(no stirring)-MeOH. This remark led us to successively achieved a bark and leaves discrimination and to assess the impact of the nature of techniques of extraction on their respective biological activities. 

**Figure 3:**
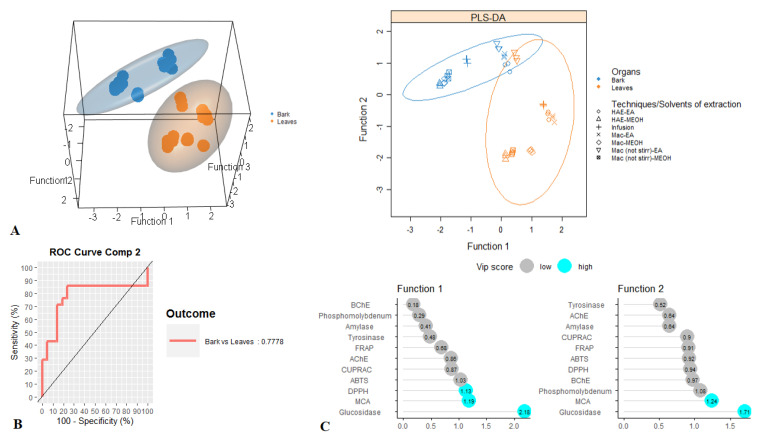
Discrimination between leaves and bark samples through PLS-DA. (**A**). Three- and two-dimensional score plots. (**B**). Area Under the Curve average using one-vs-all comparisons. (**C**). Biological activities assays explaining the largest part of the variance between the two organs.

**Figure 4:**
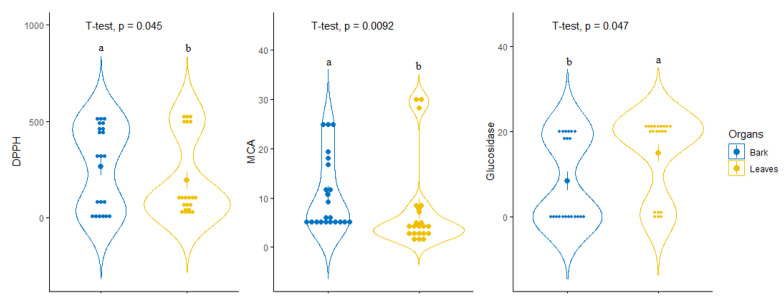
Comparison of leaves and bark taking account of the identified most discriminant biological activities.

For tentative diagnosing discrimination between the two organs, datasets derived from evaluated biological activities were submitted to PLS-discriminant analysis. Scatter plot of PLS-DA analysis, pointed to a separation of bark and leaves extracts, with satisfactory parameters, being 0.77 for AUC when taking into account the first two components (Figure 3A,B). Afterwards, the most significant assays that allowed us to separate the two organs were identified. Among all evaluated biological activities glucosidase, MCA and DPPH were found to be the most important assays for organ discrimination (Figure 3C). A comparative assessment into the consideration of these biological assays showed that the highest DPPH radical scavenging and Cu^2+^ chelating activity was observed in the stem bark samples while leaf samples exhibited important inhibitory against glucosidase compare to those of stem bark (Figure 4). 

Considering the great variations among the samples emphasized by multivariate statistics, it is important to note that the phytoconstituents recovery and in a wider the biological activities of *R. racemosa* were strongly impacted by the nature of solvents/technique of extraction used and varied greatly from one organ too another. In fact, various kinds of constituents, categorized into primary and secondary metabolites, are synthesized by the plant. Secondary metabolites such as phenolics, play a crucial role in plant functions and the interaction between plant and its environment. Further, the distribution and accumulation of secondary metabolites within the different organs, tissues and cells of plants, depend on their specific biological functions. As a result, the choice of solvent and technique for herbals phytoconstituents extraction should take into account their chemical characteristics, polarities and uneven distribution in the herbals matrix. 

### 3.5. Anti-bacterial and Anti-fungal Activities

All extracts showed anti-microbial activity within the concentration range tested (Table 5) but with wide variability in terms of potency and selectivity. The strongest inhibition was observed for leaf extracts obtained by static maceration in EA and MeOH (MIC 0.9 mg/mL, against *E. coli*) while the extract obtained by maceration of bark in methanol resulted in reducing *P. aeruginosa* growth. The differences between the activity of extracts from different plant parts do not highlight significant differences, as evidenced by the similar profile for the water infusion, HAE-MeOH and static maceration in methanol that could be considered as the extracts with a broader spectrum of activity, with MIC values lower than 1 mg/mL on all tested strains. Conversely, the EA extraction by static maceration seems to be ineffective against all tested microorganisms. Among leaf extracts, the HAE-EA results were the most promising in terms of efficacy and spectrum of activity. All strains are sensible with MIC values lower than 0.37 mg/mL. The same extracts obtained from bark give similar results, but with slightly higher MIC values against *E. coli* and *S. aureus* (0.56 mg/mL). 

All tested extracts resulted active as inhibitory of fungal growth, but huge variability was recorded between the most active (bark HAE-MEOH on *T. viride*: MIC 0.004 mg/mL) and the less active (bark HAE-EA on *A. fumigatus*: MIC 3.0 mg/mL). The strongest activity was registered for bark extracts HAE-MEOH and maceration-MEOH against *T. viride* with MIC values of 0.004 mg/mL and 0.017 mg/mL, respectively. The less sensible strain resulted *A. fumigatus* (MIC range 3–0.56 mg/mL) while all extracts actively reduce the viability of *T. viride* in the concentration range 0.004–0.75 mg/mL. Of the 14 tested extracts, 9 were able to inhibit the fungal growth at concentration 0.37 mg/mL or lower. Leaf maceration in not stirred MEOH and infusion are the extracts with a broader spectrum of activity, they inhibit the growth of all tested strains at low concentrations (MIC range 0.75–0.13 mg/mL and 0.56–0.09 mg/mL, respectively, with the only exception of higher MIC value registered for infusion on the less sensible *A. fumigatus* (Table 6). Conversely, the least promising extract as anti-fungal agents in bark HAE-EA that showed inhibition values higher than 1.5 except for the value registered against *T viride*. The MIC of 0.56 mg/mL results anyway one of the higher against this strain. The activity of leaves extracts Maceration-MEOH against *A. ochraceus* (MIC 0.18 mg/mL) and maceration (not stirred)-MEOH on *P. ochrochlorum* (MIC 0.13 mg/mL) was also notable. No general consideration is highlighted in terms of anti-fungal potency comparing the extracts from bark or leaves. Considering a cutoff at 0.75 mg mL^−1^ as an indicator for relevant anti-fungal activity, the efficacy scale, based on the number of sensible strains results leaves infusion and maceration (not stirred)-MEOH > bark maceration (not stirred)-EA and maceration (not stirred)-MeOH > bark HAE-MEOH > bark Bark-maceration–MEOH, leaves maceration-MEOH and HAE-MEOH > leaves maceration EA > bark infusion and maceration EA, leaves HAE-EA and maceration (not stirred)-EA > bark HAE-EA.

### 3.6. Bioinformatics and Docking Studies

**Figure 5:**
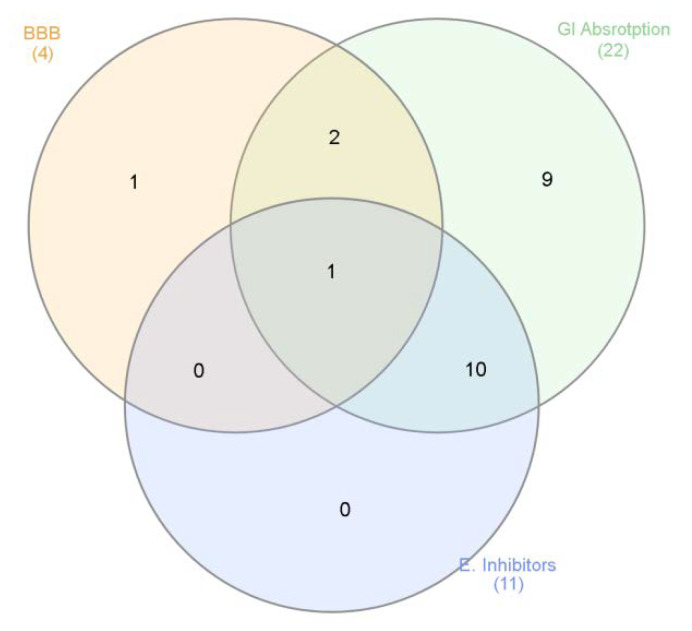
Venn diagram summarizing the criteria for selecting extracts’ phytochemicals, basing on pharmacokinetics (Gastrointestinal (GI) absorption and blood brain barrier (BBB) crossing) and biological properties (enzyme inhibition).

Finally, based on both phytochemical and biological assays, a bioinformatics study was conducted to predict the pharmacokinetics and putative targets of phytochemicals identified in the extracts. The bioinformatics evaluation was performed using SwissADME and SwissTargetPrediction software (Swiss Institute of Bioinformatics) and complete results are reported as supplementary material (Supplementary Material_Bioinformatic Folder). Specifically, the evaluation considered more than 30 phytochemicals that were identified in all analyzed and tested extracts. As expected, a wide plethora of target proteins was predicted by SwissTargetPrediction software, although with different interaction probability (range: 0.05–1 mg/mL). The phytocompounds were then classified according to their capability to be absorbed by the gastrointestinal tract (GI), to cross blood–brain barrier (BBB) and interact with the tested enzymes, namely cholinesterases, α-glucosidase, α-amylase and tyrosinase (Figure 5).

**Figure 6:**
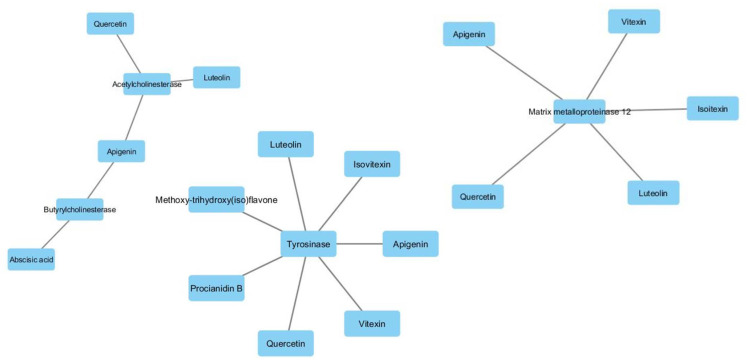
Components-targets analysis highlighting the putative interactions of luteolin, apigenin, vitexin, isovitexin, quercetin, procyanidin B and methoxy-trihydroxy(iso)flavone with tyrosinase, cholinesterases and metalloproteinase-12.

Subsequently, a network pharmacology analysis was performed via Cytoscape software to highlight the most probable putative interactions, towards tested enzymes. Although no identified phytochemical was predicted to interact with α-glucosidase and/or α-amylase, thus indicating that the inhibition of these two enzymes could be aspecific, multiple secondary metabolites present in the extracts were predicted to interact with tyrosinase and cholinesterases. Regarding the cholinesterases, the interactions predicted for abscisic acid could be of interest and consistent with the ability of this compound to cross BBB. Conversely, the lack of capability to cross BBB displayed by apigenin, luteolin and quercetin scale back their importance as anti-cholinesterase agents (Supplementary Material_Bioinformatic Folder; Figure 6). Nevertheless, these compounds, but also vitexin, isovitexin, methoxy-trihydroxy(iso)flavone and procyanidin B, were predicted to interact with tyrosinase (Figure 6). Given the anti-tyrosinase effects of the tested extracts, the predicted interactions could be therapeutically relevant against hyperpigmentation. Furthermore, isovitexin, vitexin, apigenin, luteolin and quercetin were predicted to interact with metalloproteinase-12 (MMP-12) (Figure 6). whose activity was significantly increased in skin disorders, including melanoma [25].

**Figure 7:**
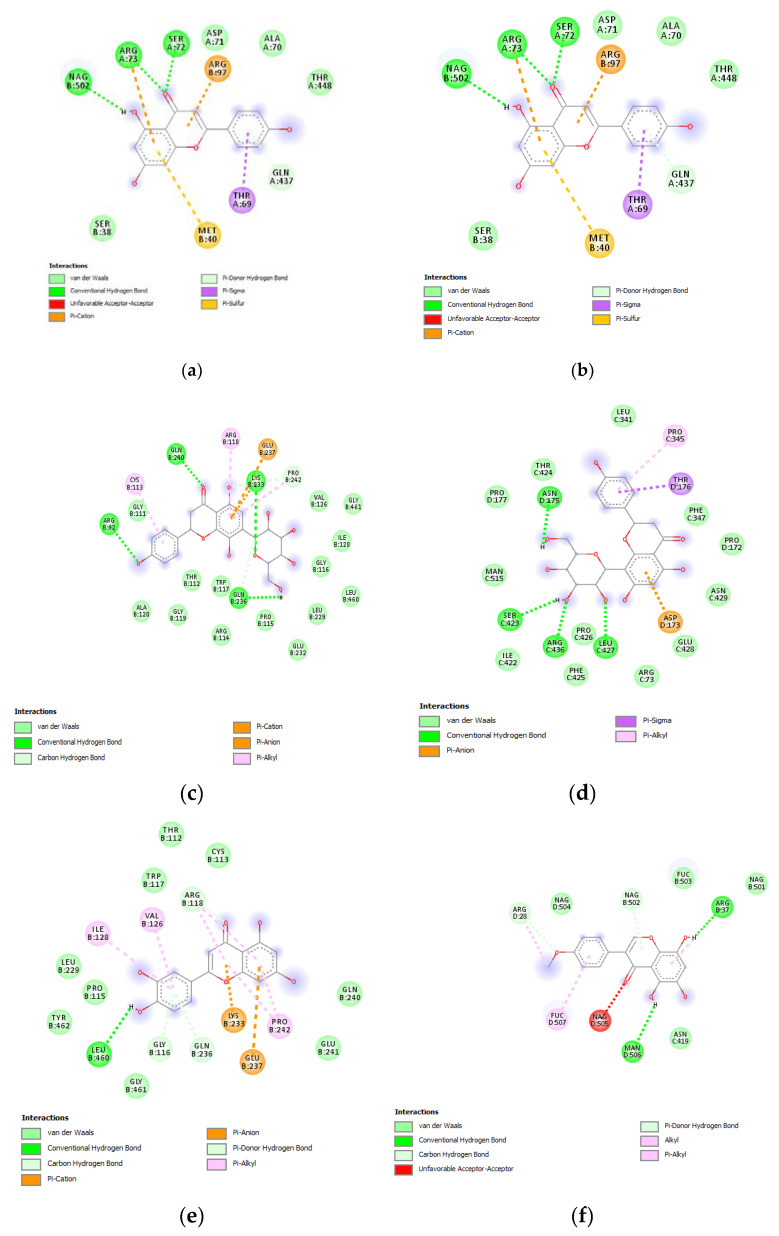

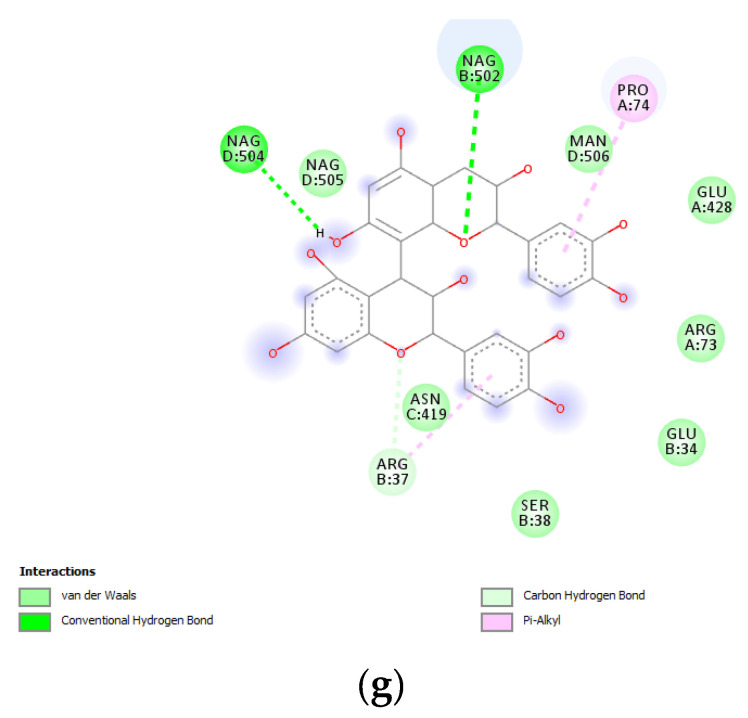
(**a**) Putative interactions between apigenin and tyrosinase (Protein Data Bank (PDB): 5M8P). Free energy of binding (ΔG) and affinity (Ki) are −6.8 kcal/mol and 10.5 µM, respectively. (**b**) Putative interactions between quercetin and tyrosinase (PDB: 5M8P). Free energy of binding (ΔG) and affinity (Ki) are −7.1 kcal/mol and 6.3 µM, respectively. (**c**) Putative interactions between isovitexin and tyrosinase (PDB: 5M8P). Free energy of binding (ΔG) and affinity (Ki) are −8.9 kcal/mol and 0.3 µM, respectively. (**d**) Putative interactions between vitexin and tyrosinase (PDB: 5M8P). Free energy of binding (ΔG) and affinity (Ki) are −9.0 kcal/mol and 0.3 µM, respectively. (**e**) Putative interactions between luteolin and tyrosinase (PDB: 5M8P). Free energy of binding (ΔG) and affinity (Ki) are −8.3 kcal/mol and 0.8 µM, respectively. (**f**) Putative interactions between methoxy-trihydroxy(iso)flavone and tyrosinase (PDB: 5M8P). Free energy of binding (ΔG) and affinity (Ki) are −6.5 kcal/mol and 17.4 µM, respectively. (**g**) Putative interactions between procyanidin B and tyrosinase (PDB: 5M8P). Free energy of binding (ΔG) and affinity (Ki) are −8.6 kcal/mol and 0.5 µM, respectively.

**Figure 8:**
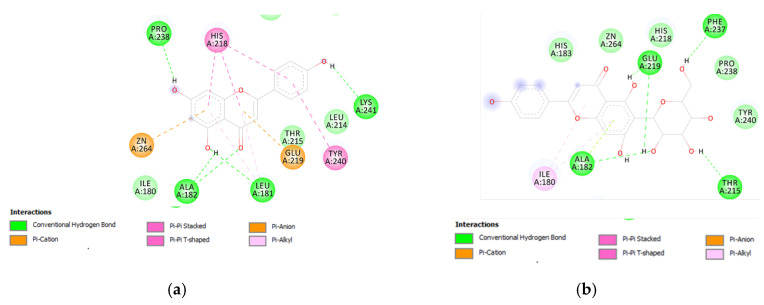

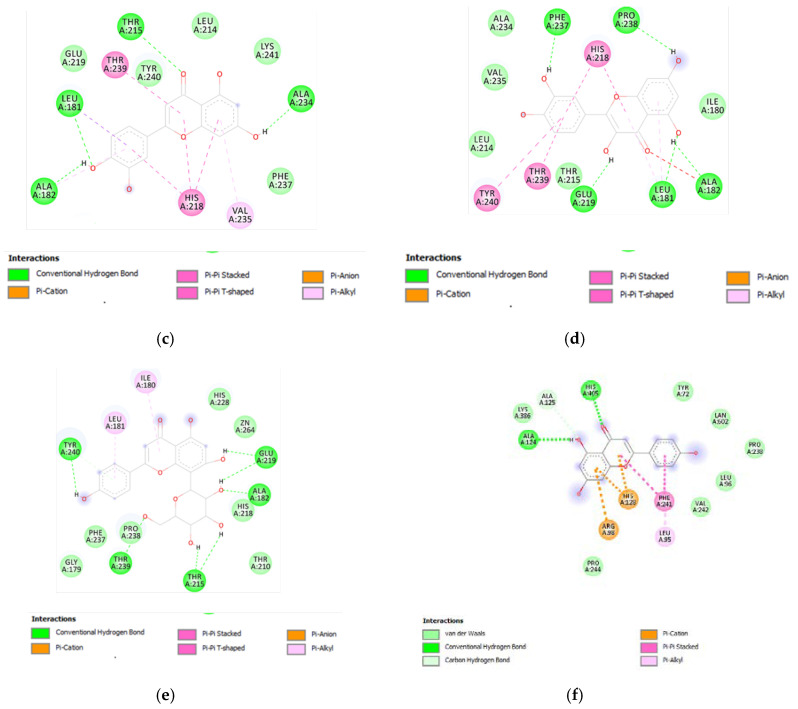
(**a**) Putative interactions between apigenin and MMP-12 (PDB: 3F17). Free energy of binding (ΔG) and affinity (Ki) are −8.3 kcal/mol and 0.8 µM, respectively. (**b**) Putative interactions between isovitexin and MMP-12 (PDB: 3F17). Free energy of binding (ΔG) and affinity (Ki) are −8.8 kcal/mol and 0.4 µM, respectively. (**c**) Putative interactions between luteolin and MMP-12 (PDB: 3F17). Free energy of binding (ΔG) and affinity (Ki) are −8.7 kcal/mol and 0.4 µM, respectively. (**d**) Putative interactions between quercetin and MMP-12 (PDB: 3F17). Free energy of binding (ΔG) and affinity (Ki) are −8.7 kcal/mol and 0.4 µM, respectively. (**e**) Putative interactions between vitexin and MMP-12 (PDB: 3F17). Free energy of binding (ΔG) and affinity (Ki) are −8.8 kcal/mol and 0.3 µM, respectively. (**f**) Putative interactions between apigenin and lanosterol-14-alpha demethylase (PDB: 4LXJ). Free energy of binding (ΔG) and affinity (Ki) are −8.8 kcal/mol and 0.4 µM, respectively.

Therefore, a docking analysis was carried out, to investigate the interactions between apigenin, quercetin, vitexin, isovitexin, methoxy-trihydroxy(iso)flavone and procyanidin B with tyrosinase and MMP-12 (Figure 7 and Figure 8).

**Figure 9:**
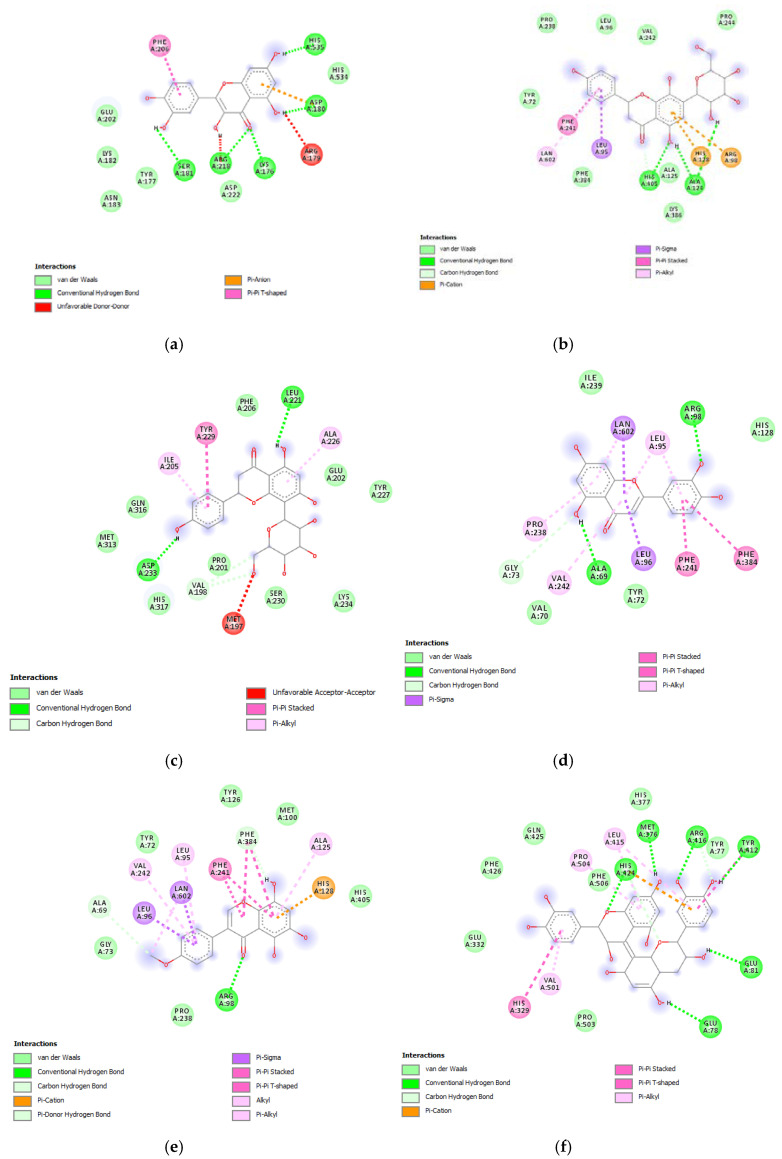
(**a**) Putative interactions between quercetin and lanosterol-14-alpha demethylase (PDB: 4LXJ). Free energy of binding (ΔG) and affinity (Ki) are −8.8 kcal/mol and 0.4 µM, respectively. (**b**) Putative interactions between isovitexin and lanosterol-14-alpha demethylase (PDB: 4LXJ). Free energy of binding (ΔG) and affinity (Ki) are −9.4 kcal/mol and 0.1 µM, respectively. (**c**) Putative interactions between vitexin and lanosterol-14-alpha demethylase (PDB: 4LXJ). Free energy of binding (ΔG) and affinity (Ki) are −9.4 kcal/mol and 0.1 µM, respectively. (**d**) Putative interactions between luteolin and lanosterol-14-alpha demethylase (PDB: 4LXJ). Free energy of binding (ΔG) and affinity (Ki) are −8.2 kcal/mol and 1 µM, respectively. (**e**) Putative interactions between methoxy-trihydroxy(iso)flavone and lanosterol-14-alpha demethylase (PDB: 4LXJ). Free energy of binding (ΔG) and affinity (Ki) are −8.7 kcal/mol and 0.4 µM, respectively. (**f**) Putative interactions between procyanidin B and lanosterol-14-alpha demethylase (PDB: 4LXJ). Free energy of binding (ΔG) and affinity (Ki) are −8.3 kcal/mol and 0.8 µM, respectively.

**Figure 10:**
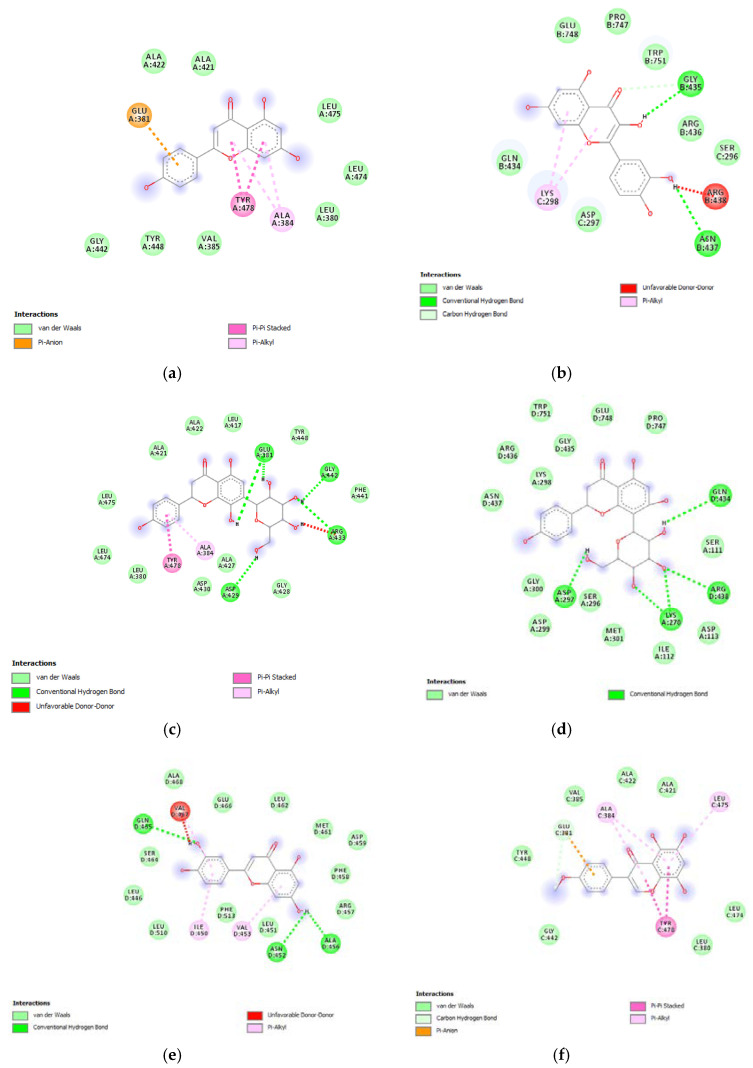

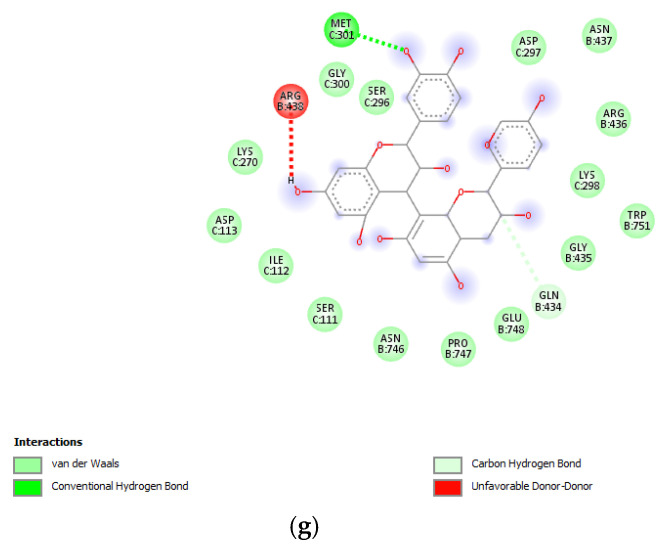
(**a**) Putative interactions between apigenin and *E. coli* gyrase (PDB: 6RKS). Free energy of binding (ΔG) and affinity (Ki) are −7.3 kcal/mol and 4.5 µM, respectively. (**b**) Putative interactions between quercetin and *E. coli* gyrase (PDB: 6RKS). Free energy of binding (ΔG) and affinity (Ki) are −7.2 kcal/mol and 5.4 µM, respectively. (**c**) Putative interactions between isovitexin and *E. coli* gyrase (PDB: 6RKS). Free energy of binding (ΔG) and affinity (Ki) are −8.4 kcal/mol and 0.7 µM, respectively. (**d**) Putative interactions between vitexin and *E. coli* gyrase (PDB: 6RKS). Free energy of binding (ΔG) and affinity (Ki) are −8.5 kcal/mol and 0.6 µM, respectively. (**e**) Putative interactions between luteolin and *E. coli* gyrase (PDB: 6RKS). Free energy of binding (ΔG) and affinity (Ki) are −7.7 kcal/mol and 2.3 µM, respectively. (**f**) Putative interactions between methoxy-trihydroxy(iso)flavone and *E. coli* gyrase (PDB: 6RKS). Free energy of binding (ΔG) and affinity (Ki) are −6.9 kcal/mol and 8.9 µM, respectively. (g) Putative interactions between procyanidin B and *E. coli* gyrase (PDB: 6RKS). Free energy of binding (ΔG) and affinity (Ki) are −8.0 kcal/mol and 1.4 µM, respectively.

Furthermore, the tested extracts were also effective towards multiple bacterial and fungi strains involved in skin disorders [26,27,28,29,30,31,32,33,34], thus highlighting the importance to deepen our knowledge about the mechanisms underlying the aforementioned inhibitory effects. In this context, these compounds were docked towards DNA-gyrase and 14-lanosterol-α-demethylase, which are key enzymes in the metabolism of bacteria and fungi, respectively [35,36]. The compounds were also docked against DNA-gyrase and 14-lanosterol-α-dmethylase. The results of the docking study are reported in Figure 9 and Figure 10, including ΔG and Ki values.

Overall, the results of tyrosinase inhibition assay and bioinformatic analyses suggest the extracts of *R. racemosa* as sources of phytochemicals with promising applications in inflammatory and infectious skin disorders. Among the tested compounds, isovitexin could be the most promising phytochemical, with Ki values calculated towards the selected enzymes, in the nanomolar range between −9.4 and −8.4 kcal/mol, corresponding to submicromolar Ki values. This could be related, at least partially, to the higher number of favorable non-covalent bonds predicted by docking analysis.

## 4. Discussion

Data collected in this present study highlighted the key role of extraction techniques and solvent choice in the quest of phytochemicals from *R. racemosa*. Overall it was demonstrated that methanol was a better extraction medium irrespective of the extraction technique used. In terms of antioxidant assays, both methanolic leaf and bark extracts exhibited remarkable reducing potential. Plants are considered as major biological factories for anti-oxidants having the potential to scavenge and detoxify reactive oxygen species (ROS) [37]. ROS are the major precursors of the development of chronic diseases namely diabetes mellitus, cancer, inflammatory disorders, cardiovascular diseases among others [38,39]. On this note, it is acknowledged that anti-oxidants are essential in preventing ROS from initiating diseases. Interestingly, several phytochemicals are reported to possess potent antioxidant capacities due to their ability to donate electrons and/or chelate transition metals [40].

Furthermore, our study also explored the enzymatic inhibitory activities of the extracts. Medicinal plants are abounded with secondary metabolites having broad-spectrum of enzyme inhibitory potentials [41,42]. It is thus of paramount importance to continue searching for inhibitors to tune down activities of enzymes which subsequently prevent initiation and/or progression of diseases. Hence, the focus of numerous researchers has now geared towards plants to successfully develop potential enzyme inhibitors. Herein, the enzymatic inhibitory effects of the mangrove plant, *R. racemosa* were investigated against key enzymes responsible for developing chronic diseases such as diabetes mellitus type II, neurodegenerative disorders and skin hyperpigmentation. As far as our literature search could reach, this report is second-to-none in appraising the enzymatic inhibitory potential of *R. racemosa*. It is of noteworthy interest to highlight that the highest enzyme inhibitory effects were observed against tyrosinase. In this context, bioinformatics analysis predicted putative interactions between tyrosinase and multiple secondary metabolites including apigenin, luteolin, vitexin, isovitexin, procyanidin B, quercetin and methoxy-trihydroxy(iso)flavone. The same compounds were also docked against lanosterol-14α-demethylase and *E. Coli* DNA gyrase, yielding affinities in the submicromolar–micromolar range that further support the observed anti-microbial effects exerted by the extracts. Regarding the antibacterial activity, the selective efficacy against *E. coli* exerted by static maceration extract could support the traditional use of mangrove as a source of products to treat intestinal disorders, such as diarrhea [43]. Similarly, the same extracts, also derived from bark are potential candidates to treat infections caused by *S. aureus* that are frequently related to skin diseases such as abscesses [26]. The extracts were also active against anti-mycotic agents. *T. viride*, which is well known for its antagonistic ability towards plant pathogenic fungi and received considerable attention as a biocontrol agent of soil-borne plant pathogens [44]. The production of secondary metabolites by *Trichoderma* strains also shows a great variety and applicative potential in medicine as anti-microbial and anti-fungal agents and as well in agriculture, where the main application is as biocontrol and biofertilizer. In terms of strains susceptibility, the effects on *A. ochraceus,* one of the main contaminants of foodstuff such as rice, wheat or maize [45], the results were interesting.

## 5. Conclusions

As a conclusion, the leaves and barks of *R. racemosa* represent a potential storehouse of secondary metabolites with promising biological activities for the development of novel pharmacophores. However, further investigation is required to evaluate the toxicity, efficacy and bioavailability of the observed pharmacological effects.

## Figures and Tables

**Table 1:** Total bioactive compounds (phenolic (TPC), flavonoid (TFC), phenolic acid (TPAC) and flavonol (TFlv) of the tested extracts *.

Parts	Methods-Solvents	TPC (mg GAE/g)	TFC (mg RE/g)	TPAC (mg CAE/g)	TFlv (mg CE/g)
Leaves	infusion	83.85 ± 1.12 ^f^	17.22 ± 0.30 ^e^	26.40 ± 0.77 ^d^	1.66 ± 0.01 ^ij^
	HAE-EA	50.17 ± 0.57 ^g^	24.06 ± 2.09 ^cd^	nd	8.28 ± 0.03 ^f^
	HAE-MEOH	217.21 ± 0.69 ^a^	33.10 ± 1.78 ^b^	58.50 ± 2.03 ^a^	62.43 ± 0.41 ^b^
	Maceration EA	35.44 ±0.32 ^h^	25.57 ± 0.75 ^c^	nd	2.92 ± 0.06 ^hi^
	Maceration-MEOH	198.57 ± 5.51 ^c^	38.45 ± 0.75 ^a^	49.69 ± 2.35 ^b^	60.42 ± 0.36 ^c^
	Maceration (not stirred)-EA	24.27 ± 0.40 ^j^	22.20 ± 0.35 ^d^	nd	1.46 ± 0.02 ^ij^
	Maceration (not stirred)-MEOH	191.74 ± 0.64 ^d^	38.07 ± 0.60 ^a^	40.39 ± 7.11 ^c^	58.11 ± 0.98 ^d^
Bark	infusion	145.15 ± 0.39 ^e^	15.01 ± 1.76 ^ef^	61.50 ± 1.65 ^a^	5.27 ± 0.08 ^g^
	HAE-EA	30.34 ± 0.33 ^i^	7.96 ± 0.10 ^i^	nd	3.81 ± 0.02 ^gh^
	HAE-MEOH	210.00 ± 1.28 ^b^	9.00 ± 0.52 ^hi^	47.73 ± 0.93 ^b^	66.17 ± 1.40 ^a^
	Maceration EA	30.84 ± 0.18 ^hi^	8.81 ± 0.20 ^hi^	nd	2.29 ± 0.06 ^hij^
	Maceration-MEOH	201.73 ± 0.43 ^c^	13.64 ± 0.79 ^fg^	34.04 ± 0.41 ^c^	63.21 ± 1.20 ^b^
	Maceration (not stirred)-EA	28.51 ± 0.26 ^ij^	10.96 ± 0.45 ^gh^	nd	0.73 ± 0.01 ^j^
	Maceration (not stirred)-MEOH	192.92 ± 0.74 ^d^	12.52 ± 0.15 ^fg^	26.70 ± 2.74 ^d^	11.59 ± 0.09 ^e^

* Values are reported as mean ± S.D. GAE: Gallic acid equivalent; RE: Rutin equivalent; CAE: Caffeic acid equivalent; CE: Catechin equivalent; nd: not detected; HAE: Homogenizer assisted extraction; EA: Ethyl acetate; MEOH: Methanol. Different letters (a–j) indicate significant differences in the extracts (*p* < 0.05).

**Table 2:** Chemical composition of the tested extracts.

No.	Name	Formula	Leaves Infusion	Leaves HAE-EA	Leaves HAE-MeOH	Bark Infusion	Bark HAE-EA	Bark HAE-MeOH
1	Gallic acid (3,4,5-Trihydroxybenzoic acid)	C_7_H_6_O_5_	+	−	−	+	−	+
2	Protocatechuic acid (3,4-Dihydroxybenzoic acid)	C_7_H_6_O_4_	+	+	+	+	+	+
3	Pantothenic acid	C_9_H_17_NO_5_	−	−	−	+	−	+
4	Neochlorogenic acid (5-*O*-Caffeoylquinic acid)	C_16_H18O9	+	+	+	+	+	+
5	Prodelphinidin C	C_30_H_26_O_13_	−	−	−	−	−	+
6	Hydroxybenzaldehyde	C_7_H_6_O_2_	+	+	+	−	−	−
7	3-*O*-(4-Coumaroyl)quinic acid	C_16_H_18_O_8_	+	+	+	+	+	+
8 ^1^	Catechin	C_15_H_1_4O_6_	+	−	+	+	+	+
9	Esculetin (6,7-Dihydroxycoumarin)	C_9_H_6_O_4_	+	+	+	+	+	+
10 ^1^	Chlorogenic acid (3-*O*-Caffeoylquinic acid)	C_16_H_18_O_9_	+	+	+	+	+	+
11	3-*O*-Feruloylquinic acid	C_17_H_20_O_9_	+	−	+	+	−	+
12	Caffeic acid	C_9_H_8_O_4_	+	+	+	+	+	+
13	Procyanidin B	C_30_H_26_O_12_	+	+	+	+	+	+
14	Cinchonain II isomer 1	C_39_H_32_O_15_	+	−	+	+	−	+
15	Chryptochlorogenic acid (4-*O*-Caffeoylquinic acid)	C_16_H_18_O_9_	+	+	+	+	+	+
16	Cinchonain II isomer 2	C_39_H_32_O_15_	+	−	+	+	−	+
17	2-Oxindole	C_8_H_7_NO	+	+	+	+	+	+
18	Procyanidin C	C_45_H_38_O_18_	+	+	+	+	+	+
19	5-*O*-(4-Coumaroyl)quinic acid	C_16_H_18_O_8_	+	+	+	+	−	+
20 ^1^	Epicatechin	C_15_H_14_O_6_	+	+	+	+	+	+
21	Cinchonain II isomer 3	C_39_H_32_O_15_	+	−	+	−	−	−
22	4-*O*-(4-Coumaroyl)quinic acid	C_16_H_18_O_8_	+	−	+	+	−	+
23	Cinchonain II isomer 4	C_39_H_32_O_15_	−	−	+	−	−	−
24	Antiarol (3,4,5-Trimethoxyphenol)	C_9_H_12_O_4_	+	+	+	+	+	+
25	5-*O*-Feruloylquinic acid	C_17_H_20_O_9_	+	−	+	+	−	−
26 ^1^	4-Coumaric acid	C_9_H_8_O_3_	+	+	+	+	+	+
27	Tuberonic acid or 12-Hydroxyjasmonic acid	C_12_H_18_O_4_	+	+	+	+	+	+
28	4-*O*-Feruloylquinic acid	C_17_H_20_O_9_	+	−	+	+	-	+
29	Riboflavin	C_17_H_20_N_4_O_6_	+	−	+	+	−	+
30	Cinchonain I isomer 1	C_24_H_20_O_9_	−	−	+	−	+	+
31	Naringenin-*C*-hexoside isomer 1	C_21_H_22_O_10_	+	−	+	+	−	+
32 ^1^	Taxifolin (Dihydroquercetin)	C_15_H_12_O_7_	+	+	+	+	+	+
33	Quercetin-*O*-rhamnosyldihexoside	C_33_H_40_O_21_	+	+	+	+	+	+
34	Naringenin-*C*-hexoside isomer 2	C_21_H_22_O_10_	+	−	+	+	−	+
35	Quercetin-*O*-hexosylhexoside	C_27_H_30_O_17_	+	+	+	+	+	+
36	Naringenin-*C*-hexoside isomer 3	C_21_H_22_O_10_	+	−	+	+	−	+
37	Quercetin-*O*-dirhamnosylhexoside	C_33_H_40_O_20_	+	+	+	+	−	+
38	Hexahydroxy(iso)flavone-*O*-hexoside	C_21_H_20_O_13_	+	−	+	+	−	+
39	Hexahydroxy(iso)flavone-*O*-rhamnosylhexoside	C_27_H_30_O_17_	+	+	+	+	+	+
40	Cinchonain I isomer 2	C_24_H_20_O_9_	−	−	+	−	+	+
41	Cinchonain I isomer 3	C_24_H_20_O_9_	−	−	+	−	+	+
42 ^1^	Vitexin (Apigenin-8-*C*-glucoside)	C_21_H_20_O_10_	+	+	+	+	+	+
43	Kaempferol-*O*-dirhamnosylhexoside	C_33_H_40_O_19_	+	+	+	−	−	−
44	Isovitexin (Apigenin-6-*C*-glucoside)	C_21_H_20_O_10_	+	+	+	+	+	+
45 ^1^	Naringin (Naringenin-7-*O*-neohesperidoside)	C_27_H_32_O_14_	+	−	+	+	+	+
46 ^1^	Isoquercitrin (Quercetin-3-*O*-glucoside)	C_21_H_20_O_12_	−	−	+	+	+	+
47 ^1^	Rutin (Quercetin-3-*O*-rutinoside)	C_27_H_30_O_16_	+	+	+	+	+	+
48	Quercetin-*O*-pentoside	C_20_H_18_O_11_	+	+	+	−	−	−
49	Astragalin (Kaempferol-3-*O*-glucoside)	C_21_H_20_O_11_	+	+	+	−	−	−
50	Kaempferol-3-*O*-rutinoside (Nicotiflorin)	C_27_H_30_O_15_	+	+	+	+	+	+
51	Cinchonain I isomer 4	C_24_H_20_O_9_	−	−	+	−	+	+
52 ^1^	Eriodictyol (3′,4′,5,7-Tetrahydroxyflavanone)	C_15_H12O_6_	+	+	+	+	+	+
53 ^1^	Isorhamnetin-3-*O*-glucoside	C_22_H_22_O_12_	−	−	+	+	−	−
54	Tetrahydroxy(iso)flavone-*O*-rhamnosylhexoside	C_28_H_32_O_16_	+	+	+	+	+	+
55	Abscisic acid	C_15_H_20_O_4_	+	+	+	+	+	+
56 ^1^	Quercetin (3,3’,4’,5,7-Pentahydroxyflavone)	C_15_H_10_O_7_	+	+	+	+	+	+
57 ^1^	Naringenin (4’,5,7-Trihydroxyflavanone)	C_15_H_12_O_5_	+	+	+	+	+	+
58 ^1^	Luteolin (3’,4’,5,7-Tetrahydroxyflavone)	C_15_H_10_O_6_	+	+	+	+	+	+
59 ^1^	Kaempferol (3,4’,5,7-Tetrahydroxyflavone)	C_15_H_10_O_6_	+	+	+	−	−	−
60 ^1^	Apigenin (4’,5,7-Trihydroxyflavone)	C_15_H_10_O_5_	−	+	+	+	+	+
61 ^1^	Isorhamnetin (3’-Methoxy-3,4’,5,7-tetrahydroxyflavone)	C_16_H_12_O_7_	−	+	+	−	+	−
62	Methoxy-trihydroxy(iso)flavone	C_16_H_12_O_6_	−	+	+	−	+	+
63	Pinocembrin (5,7-Dihydroxyflavanone)	C_15_H_12_O_4_	−	+	+	−	−	−
64	Tetrahydroxyxanthone	C_13_H_8_O_6_	−	−	−	+	−	−
65	Tetrahydroxyxanthone isomer 1	C_13_H_8_O_6_	−	−	−	−	−	+
66	Tetrahydroxyxanthone isomer 2	C_13_H_8_O_6_	−	−	−	−	−	+
67	Luteolin-7-*O*-glucoside (Cynaroside)	C_21_H_20_O_11_	−	−	−	+	−	+
68	Luteolin-7-*O*-rutinoside (Scolymoside)	C_27_H_30_O_15_	−	−	−	+	−	+
69	Methoxy-tetrahydroxy(iso)flavone	C_15_H_10_O_5_	−	−	−	+	−	−
70	Methoxy-trihydroxy(iso)flavone isomer 1	C_16_H_12_O_6_	−	−	−	+	−	−
71	Methoxy-trihydroxy(iso)flavone isomer 2	C_16_H_12_O_6_	−	−	−	+	−	−

+: present; − absent. ^1^ Confirmed by standard

**Table 3:** Antioxidant properties of the tested extracts *.

Parts	Methods-Solvents	DPPH (mg TE/g)	ABTS (mg TE/g)	CUPRAC (mg TE/g)	FRAP (mg TE/g)	Metal chelating (mg EDTAE/g)	Phosphomolybdenum (mmol TE/g)
Leaves	infusion	101.26 ± 0.24 ^fg^	144.25 ± 3.10 ^e^	315.43 ± 5.58 ^g^	200.02 ± 4.73 ^f^	8.08 ± 0.76 ^e^	2.15 ± 0.11 ^d^
	HAE-EA	67.69 ± 5.49 ^h^	89.09 ± 1.99 ^f^	193.20 ± 3.83 ^h^	77.29 ± 1.16 ^g^	1.95 ± 0.49 ^h^	2.12 ± 0.29 ^d^
	HAE-MEOH	525.84 ± 1.37 ^a^	600.84 ± 13.60 ^a^	1047.10 ± 9.45 ^b^	544.76 ± 11.89 ^b^	4.50 ± 0.53 ^fg^	4.82 ± 0.42 ^a^
	Maceration EA	20.97 ± 0.28 ^ij^	32.41 ± 6.38 ^g^	124.15 ± 2.58 ^i^	50.25 ± 1.91 ^hi^	2.32 ± 0.50 ^gh^	2.17 ± 0.08 ^d^
	Maceration-MEOH	105.56 ± 0.06 ^f^	159.42 ± 0.12 ^e^	753.22 ± 4.81 ^f^	401.16 ± 5.03 ^e^	4.31 ± 0.95 ^fg^	4.29 ± 0.20 ^abc^
	Maceration (not stirred)-EA	39.06 ± 2.14 ^i^	12.31 ± 0.29 ^hi^	84.92 ± 0.74 ^j^	40.54 ± 0.63 ^hi^	29.39 ± 1.03 ^a^	2.40 ± 0.13 ^d^
	Maceration (not stirred)-MEOH	496.94 ± 3.30 ^bc^	519.71 ± 6.78 ^c^	825.71 ± 25.99 ^e^	519.69 ± 2.96 ^c^	3.64 ± 0.31 ^fgh^	3.95 ± 0.44 ^bc^
Bark	infusion	318.53 ± 8.82 ^e^	353.91 ± 11.81 ^d^	766.83 ± 7.23 ^f^	398.10 ± 6.46 ^e^	18.06 ± 1.29 ^c^	3.63 ± 0.14 ^c^
	HAE-EA	81.08 ± 5.23 ^gh^	33.45 ± 1.66 ^g^	94.03 ± 2.12 ^j^	49.72 ± 1.12 ^hi^	5.39 ± 0.81 ^f^	1.69 ± 0.21 ^d^
	HAE-MEOH	512.37 ± 7.15 ^ab^	581.39 ± 6.42 ^a^	1129.33 ± 8.62 ^a^	633.53 ± 8.97 ^a^	5.42 ± 0.06 ^f^	4.74 ± 0.17 ^ab^
	Maceration EA	15.86 ± 1.83 ^ij^	29.36 ± 1.37 ^gh^	126.72 ± 4.57 ^j^	55.45 ± 0.32 ^h^	10.88 ± 1.41 ^d^	1.68 ± 0.15 ^d^
	Maceration-MEOH	478.39 ± 21.72 ^c^	555.13 ± 8.47 ^b^	931.13 ± 18.04 ^d^	559.27 ± 2.63 ^b^	5.61 ± 0.16 ^f^	4.51 ± 0.36 ^ab^
	Maceration (not stirred)-EA	na	7.26 ± 0.10 ^i^	74.82 ± 0.34 ^j^	37.67 ± 1.60 ^i^	24.93 ± 0.24 ^b^	2.13 ± 0.17 ^d^
	Maceration (not stirred)-MEOH	450.51 ± 15.42 ^d^	513.29 ± 8.26 ^c^	961.26 ± 1.49 ^c^	422.30 ± 12.50 ^d^	4.89 ± 0.18 ^f^	4.25 ± 0.53 ^abc^

* Values are reported as mean ± S.D. TE: Trolox equivalent; EDTAE: EDTA equivalent; na: not active. HAE: Homogenizer assisted extraction; EA: Ethyl acetate; MEOH: Methanol. Different letters (a–j) indicate significant differences in the extracts (*p* < 0.05).

**Table 4:** Enzyme inhibitory effects of the tested extracts *.

Parts	Methods-Solvents	AChE Inhibition (mg GALAE/g)	BChE Inhibition (mg GALAE/g)	Tyrosinase Inhibition (mg KAE/g)	Amylase Inhibition (mmol ACAE/g)	Glucosidase Inhibition (mmol ACAE/g)
Leaves	infusion	5.18 ± 0.08 ^d^	0.91 ± 0.07 ^d^	na	0.15 ± 0.01 ^g^	na
	HAE-EA	8.48 ± 0.21 ^ab^	2.97 ± 0.29 ^abc^	100.94 ± 1.41 ^e^	0.86 ± 0.04 ^c^	20.01 ± 0.08 ^b^
	HAE-MEOH	8.78 ± 0.07 ^a^	na	142.59 ± 1.14 ^d^	1.24 ± 0.01 ^a^	21.17 ± 0.02 ^a^
	Maceration EA	8.35 ± 0.20 ^ab^	2.19 ± 0.48 ^c^	94.19 ± 0.88 ^fg^	0.76 ± 0.02 ^de^	20.01 ± 0.24 ^b^
	Maceration-MEOH	8.62 ± 0.04 ^a^	na	146.76 ± 2.41 ^bc^	1.14 ± 0.01 ^b^	21.17 ± 0.11 ^a^
	Maceration (not stirred)-EA	8.56 ± 0.23 ^a^	3.50 ± 0.43 ^a^	96.38 ± 1.56 ^fg^	0.71 ± 0.02 ^e^	0.96 ± 0.19 ^d^
	Maceration (not stirred)-MEOH	8.56 ± 0.08 ^a^	na	146.06 ± 1.34 ^cd^	1.22 ± 0.03 ^a^	20.98 ± 0.06 ^a^
Bark	infusion	7.07 ± 0.11 ^c^	na	25.64 ± 1.13 ^h^	0.42 ± 0.04 ^f^	na
	HAE-EA	8.34 ± 0.41 ^ab^	2.52 ± 0.41 ^bc^	96.74 ± 0.95 ^f^	0.82 ± 0.01 ^cd^	20.04 ± 0.15 ^b^
	HAE-MEOH	8.76 ± 0.05 ^a^	0.59 ± 0.10 ^de^	150.75 ± 2.26 ^ab^	1.27 ± 0.02 ^a^	na
	Maceration EA	8.61 ± 0.16 ^a^	3.03 ± 0.38 ^ab^	104.13 ± 0.59 ^e^	0.69 ± 0.02 ^e^	19.87 ± 0.02 ^b^
	Maceration-MEOH	8.76 ± 0.04 ^a^	0.52 ± 0.03 ^de^	154.33 ± 1.10 ^a^	1.23 ± 0.01 ^a^	na
	Maceration (not stirred)-EA	8.04 ± 0.14 ^b^	2.19 ± 0.42 ^c^	92.22 ± 0.61 ^g^	0.75 ± 0.04 ^de^	18.48 ± 0.40 ^c^
	Maceration (not stirred)-MEOH	8.67 ± 0.01 ^a^	1.19 ± 0.27 ^d^	149.60 ± 1.98 ^bc^	1.19 ± 0.03 ^ab^	na

* Values are reported as mean ± S.D. GALAE: Galantamine equivalent; KAE: Kojic acid equivalent; ACAE: Acarbose equivalent; na: not active; HAE: Homogenizer assisted extraction; EA: Ethyl acetate; MEOH: Methanol. Different letters (a–g) indicate significant differences in the extracts (*p* < 0.05).

**Table 5:** Antibacterial activity of tested samples, results are in mg/mL.

Samples	MIC/MBC	*S.a.*	*B.c.*	*L.m.*	*P.a.*	*E.c.*	*S.t.*	*En.cl.*
Leaves- infusion	MIC	0.75	0.37	0.37	0.30	0.43	0.37	0.56
	MBC	1.50	0.75	0.75	0.43	0.87	0.75	0.75
Leaves- HAE-EA	MIC	0.75	0.75	1.12	0.43	0.30	0.56	0.75
	MBC	1.50	1.50	1.50	0.87	0.43	0.75	1.50
Leaves- HAE-MEOH	MIC	0.37	0.13	0.27	0.27	0.27	0.27	0.37
	MBC	0.75	0.18	0.37	0.37	0.37	0.37	0.75
Leaves- Maceration EA	MIC	1.12	1.12	1.12	0.30	0.43	1.12	1.12
	MBC	1.50	1.50	1.50	0.43	0.87	1.50	1.50
Leaves- Maceration-MEOH	MIC	0.37	0.27	0.56	0.27	0.18	0.56	0.56
	MBC	0.75	0.37	0.75	0.37	0.37	0.75	0.75
Leaves- Maceration (not stirred)-EA	MIC	1.50	0.75	1.12	0.18	0.09	0.75	1.12
	MBC	3.00	1.50	1.50	0.37	0.18	1.50	1.50
Leaves- Maceration (not stirred)-MEOH	MIC	0.37	0.27	0.56	0.37	0.09	0.56	0.56
	MBC	0.75	0.37	0.75	0.75	0.18	0.75	0.75
Bark- infusion	MIC	0.56	0.27	0.75	0.75	0.18	0.37	0.37
	MBC	0.75	0.37	1.50	1.50	0.37	0.75	0.75
Bark- HAE-EA	MIC	0.75	0.56	0.75	0.65	0.75	0.75	1.50
	MBC	1.50	0.75	1.50	0.87	1.50	1.50	3.00
Bark- HAE-MEOH	MIC	0.56	0.18	0.37	0.37	0.56	0.37	0.37
	MBC	0.75	0.37	0.75	0.75	0.75	0.75	0.75
Bark- Maceration EA	MIC	0.56	0.18	0.75	0.75	0.56	1.12	0.75
	MBC	0.75	0.37	1.5	1.50	0.75	1.50	1.50
Bark- Maceration-MEOH	MIC	0.37	0.27	0.75	0.75	0.27	1.12	1.12
	MBC	0.75	0.37	1.50	1.50	0.37	1.50	1.50
Bark- Maceration (not stirred)-EA	MIC	2.25	2.25	1.50	1.50	2.25	1.12	1.12
	MBC	3.00	3.00	3.00	3.00	3.00	1.50	1.50
Bark- Maceration (not stirred)-MEOH	MIC	0.27	0.27	0.56	0.09	0.56	0.56	0.37
	MBC	0.37	0.37	0.75	0.18	0.75	0.75	0.75
Streptomycin	MIC	0.10	0.025	0.15	0.025	0.10	0.10	0.025
	MBC	0.20	0.05	0.30	0.05	0.20	0.20	0.05
Ampicillin	MIC	0.10	0.10	0.15	0.05	0.15	0.10	0.10
	MBC	0.15	0.15	0.30	0.10	0.20	0.20	0.15

*Escherichia coli* (*E.c.*), *Pseudomonas aeruginosa* (*P.a.*), *Salmonella typhimurium* (*S.t.*), *Listeria monocytogenes* (*L.m.*), *Enterobacter cloacae* (*E.c.*), *Bacillus cereus* (*B.c.*), *Micrococcus luteus* (*M.l.*), *Enterobacter cloacae* (*En.cl.*) and *Staphylococcus aureus* (*S.a.*).

**Table 6:** Anti-fungal activity of tested samples, results are in mg/mL.

Samples	MIC/MFC	*A.f.*	*A.o.*	*A.n.*	*A.v.*	*P.o.*	*P.f.*	*T.v.*	*P.v.c.*
Leaves- infusion	MIC	2.25	0.37	0.37	0.37	0.09	0.18	0.27	0.56
	MFC	3.00	0.75	0.75	0.75	0.18	0.37	0.37	0.75
Leaves- HAE-EA	MIC	1.12	0.75	1.50	0.75	0.37	0.75	0.75	1.50
	MFC	1.50	1.50	3.00	1.50	0.75	1.50	1.50	3.00
Leaves- HAE-MEOH	MIC	0.56	0.75	0.75	0.27	1.12	0.37	0.22	0.75
	MFC	1.12	1.50	1.50	0.37	1.50	0.75	0.44	1.50
Leaves- Maceration EA	MIC	1.50	0.37	1.50	0.75	0.37	1.12	0.37	1.50
	MFC	3.00	0.75	3.00	1.50	0.75	1.50	0.75	3.00
Leaves- Maceration-MEOH	MIC	0.75	0.18	0.75	0.37	0.75	0.37	0.22	0.75
	MFC	1.50	0.37	1.50	0.75	1.50	0.75	0.44	1.50
Leaves- Maceration (not stirred)-EA	MIC	1.50	0.75	1.50	0.37	1.12	0.75	0.37	1.12
	MFC	3.00	1.50	3.00	0.75	1.50	1.50	0.75	1.50
Leaves- Maceration (not stirred)-MEOH	MIC	0.75	0.18	0.27	0.37	0.13	0.27	0.37	0.37
	MFC	1.50	0.37	0.37	0.75	0.18	0.37	0.75	0.75
Bark- infusion	MIC	0.75	0.37	0.75	1.12	1.12	0.75	0.27	0.84
	MFC	1.50	0.75	1.50	1.50	1.15	1.50	0.37	1.12
Bark- HAE-EA	MIC	3.00	3.00	3.00	1.50	2.25	3.00	0.56	3.00
	MFC	6.00	6.00	6.00	3.00	3.00	6.00	0.75	6.00
Bark- HAE-MEOH	MIC	0.75	0.37	0.75	1.50	0.75	0.37	0.004	0.56
	MFC	1.50	0.75	1.50	3.00	1.50	0.75	0.008	0.75
Bark- Maceration EA	MIC	1.12	0.37	0.75	0.75	0.75	1.12	0.27	0.75
	MFC	1.50	0.75	1.50	1.50	1.50	1.50	0.37	1.50
Bark- Maceration-MEOH	MIC	1.12	0.37	0.75	0.75	0.27	0.27	0.017	1.12
	MFC	1.50	0.75	1.50	1.50	0.37	0.37	0.025	1.15
Bark- Maceration (not stirred)-EA	MIC	0.75	0.37	0.56	0.37	0.56	0.56	0.27	0.75
	MFC	1.50	0.75	0.75	0.75	0.75	0.75	0.37	1.50
Bark- Maceration (not stirred)-MEOH	MIC	0.75	0.37	0.56	0.37	0.56	0.37	0.18	1.12
	MFC	1.50	0.75	0.75	0.75	0.75	1.50	0.37	1.15
Bifonazole	MIC	0.15	0.15	0.15	0.1	0.20	0.2	0.15	0.1
	MFC	0.2	0.20	0.2	0.2	0.25	0.25	0.2	0.2
Ketoconazole	MIC	0.2	0.15	0.2	0.2	1.00	0.2	1	0.2
	MFC	0.5	0.20	0.5	0.5	1.50	0.5	1.5	0.3

*Aspergillus fumigatus* (*A.f.*), *Aspergillus niger* (*A.n.*), *Trichoderma viride* (*T.v.*), *Penicillium funiculosum* (*P.f.*), *Penicillium ochrochloron* (*P.o*) and *Penicillium verrucosum* (*P.v.*).

## Supplementary Materials

The following are available online at https://www.mdpi.com/2076-3921/9/6/533/s1.
